# Intestinal Tuberculosis

**Published:** 1913-02

**Authors:** 


Intestinal Tuberculosis.—S. G. Gant of New York, states the frequence of different forms as follows (N. Y. State Jour., September, 1911:

| | Per cent. |
|---|---|
| (a) Enteric or Superficial Ulcerative type | 53.9 |
| (b) Entero-peritoneal or Deep Ulcerative type | 26.9 |
| (c) Hyperplastic or Tumor-forming type | 9.6 |
| (d) Fibro-sclerotic type (per cent. not given.) | |
| (e) Peritoneal type | 7.7 |
| (f) Glandular type | 1.1 |

